# Effects of sweetener sucralose on diet preference, growth performance and hematological and biochemical parameters of weaned piglets

**DOI:** 10.5713/ajas.18.0863

**Published:** 2019-05-28

**Authors:** Wenwei Zhang, Holden He, Limin Gong, Wenqing Lai, Bing Dong, Liying Zhang

**Affiliations:** 1 State Key Laboratory of Animal Nutrition, Ministry of Agriculture Feed Centre, China Agricultural University, Beijing 100193, China; 2 Nanjing Jinhe Yikang Biotechnology, Jiangbei New Area, Nanjing 210043, China

**Keywords:** Sucralose, Weaned Piglet, Diet Preference, Growth Performance, Hematological Parameter

## Abstract

**Objective:**

Two experiments were conducted to investigate the effects of dietary sucralose on diet preference and growth performance of weaned piglets, and a third experiment was a 28-d safety study to examine if high-dose sucralose could affect the health state of weaned piglets.

**Methods:**

In experiment one, 48 piglets had free access to a corn-soybean based diet and the same diet supplemented with 150 mg/kg sucralose for 15 d. In experiment two, 180 piglets were blocked into 5 treatments with 6 replications. They were fed basal diets supplemented with 0, 75, 150, 225, and 300 mg/kg sucralose for 28 days. In experiment three, 108 piglets were randomly assigned to 3 treatments and fed diets supplemented with 0, 150 (suitable level), and 1,500 (ten-fold suitable level) mg/kg sucralose for 28 d.

**Results:**

The experiment 1 showed that piglets preferred (p<0.05) diets containing sucralose during experimental period. In experiment 2, piglets fed a diet supplemented with 150 mg/kg sucralose had a higher average daily gain (ADG) and average daily feed intake (ADFI) than pigs in the control group and other treatment groups during the experiment period. The concentrations of sucralose over 150 mg/kg may decrease feed intake. However, no difference in feed conversion ratio was observed. In experiment 3, piglets fed diet supplemented with 150 mg/kg sucralose had a higher ADG and ADFI than that of pigs in the control group and 1,500 mg/kg treatment groups during the experiment period. Clinical blood metabolites, organ index and histological morphology were not significantly different between sucralose treatments.

**Conclusion:**

Sucralose can promote feed intake and thereby improve growth performance of weaned piglets. Moreover, inclusion of 1,500 mg/kg sucralose was demonstrated to have no observed adverse effects. Supplementing 150 mg/kg sucralose for weaned piglets is recommended in this study.

## INTRODUCTION

Weaning stress causes many problems for piglets, such as low feed intake, poor growth performance, high morbidity and high mortality [1]. The decrease in feed intake also results in malnutrition, reduction in transient growth rate and intestinal dysfunction [2]. Enhancing feed intake in the weaned pig will prevent villous atrophy, reducing post-weaning diarrhea and stimulate growth. Therefore, it is critical to improve feed intake. Efforts can be made to enhance feed intake such as optimizing feed formula, improving diet digestibility, providing palatable feedstuffs, flavors and taste enhancers [3]. Sweetened feed provided during weanling period can reduce post weaned stress and can benefit feed intake recover in piglets [4]. Some studies have shown that diets supplemented with sweeteners, such as sucrose, lactose, glucose [5], or high-intensity artificial sweeteners, such as saccharin [6] and neotame [7], increase feed intake and weight in newly weaned pigs. Kennedy and Baldwin [8] reported sucrose was more attractive than glucose or lactose to pigs. Some artificial sweeteners have bitter aftertaste or metallic odors, or the sweet characteristic is somewhat different from that of sucrose [9–11]. Sweet taste is a natural preference in humans and pigs [12]. Since the number of taste buds in the tongue of pigs are 3 to 4 times more than that of humans [13], pigs are more sensitive to sweet taste. Thus, sweeteners, which have similar taste to sucrose, may be better suited to improve feed intake in the early post-weaning period.

Sucralose (1,6-dichloro-1,6-dideoxy-β-D-fruct-ofuranosyl-4-chloro-4-deoxy-x-D-galactopyranoside) is a non-nutritive artificial sweetener, which is made from sucrose in a five-step process. Through the process, three atoms of chlorine are selective substitutes for three hydroxyl groups in the sucrose molecule [14] and thereby sucralose is 600 times sweeter than sucrose [15]. Extensive databases of studies on mice, rats, rabbits and dogs shows sucralose is non-teratogenic, non-carcinogenic and non-mutagenic and does not produce reproductive toxicity [16]. Due to its sugar-like taste, lack of bitter aftertaste, stability at high temperature, and long shelf-life [14,17], sucralose has been widely used as a sweetener and flavor enhancer in the food industry [18]. Hence, sucralose could be an ideal feed additive for weaned piglets to stimulate feed intake and consequently improve growth performance of weaned piglets. However, to our knowledge, few studies have been conducted on the effects of sucralose on piglets. Therefore, the objective of this study was to investigate the effects of dietary inclusion of sucralose on diet preference, growth performance, and hematological and biochemical parameters of weaned piglets.

## MATERIALS AND METHODS

### Animal care

This study was approved by the Institution Animal Care and Use Committee of China Agricultural University (CAU2017 0801-3).

### Animals, diets and experimental design

All piglets had access to water and creep feed before weaning. Three experiments were performed at the Swine Nutrition Research Center of the National Feed Engineering Technology Research Center (Chengde, Hebei, China). Weaned piglets were raised in an environmentally controlled house with slatted floor pens (1.2×2.0 m^2^). Each pen was equipped with a nipple drinker and two stainless steel feeders for experiment 1, and one feeder for experiments 2 and 3. Pigs were allowed *ad libitum* access to feed and water. The basal diet was a corn-soybean based diet formulated to meet or exceed the NRC [19] recommendations for weaned piglets (Table 1).

#### Experiment 1

Experiment 1 was designed to determine if supplementary sucralose could affect diet preference of weaned piglets. Forty-eight weaned piglets (8.90±0.09 kg), were blocked by body weight (BW), ancestry and gender and divided into 1 of 2 dietary treatments with 8 pens per treatment and 6 pigs (3 male and 3 female) per pen. Piglets from the same sow are distributed equally to each treatment. The diet preference protocol was derived from the Richter-type test called the two-bottle preference test [20]. Each feeder contained one experimental diet: the corn-soybean basal diet (Phase I in Table 1) and the same diet supplemented with 150 mg/kg sucralose. The experiment lasted for 15 d, including a 5-d adaptation period and a 10-d experimental period. On the first day of adaption period, all pigs were fed creep feed. On d 2, they were fed mixed feed with 1:1 ratio of creep feed to control diet. And from d 3 to 5, all pigs were fed control diet. The amount of feed consumed was determined daily to calculate feed intake and diet preference percentage. Diet preference was determined using the following equation:

Diet preference percentage=Intake of one diet (g)/Total feed intake (g)×100%

#### Experiment 2

This experiment was conducted to evaluate the effects of sucralose supplementation on performance of weaned piglets. A total of 180 weaned piglets (7.95±0.17 kg), were blocked by BW, ancestry and gender and divided into 1 of 5 dietary treatments with 6 pens per treatment and 6 pigs (3 male and 3 female) per pen. Piglets from the same sow are distributed equally to each treatment. The experimental diets were a basal diet (Table 1) supplemented with 0, 75, 150, 225, or 300 mg/kg sucralose. The experiment included two phases (d 0 to 14 and 15 to 28) lasting for 28 days. Pigs and feeders were weighed on d 0, 14, and 28 to calculate average daily gain (ADG), average daily feed intake (ADFI), and feed efficiency (G:F).

#### Experiment 3

Experiment 3 was conducted to determine the effects of sucralose on hematological and biochemical parameters of weaned piglets. A total of 108 weaned piglets (7.97±0.18 kg), were blocked by BW, ancestry and gender and divided into 1 of 3 dietary treatments with 6 pens per treatment and 6 pigs (3 male and 3 female) per pen. Piglets from the same sow are distributed equally to each treatment. Pigs were fed the basal diet (Table 1) or the same diet supplemented with 150 or 1,500 mg/kg sucralose. The experiment was conducted over 28 days. Pigs and feeders were weighed on d 0, 14, and 28 to calculate ADG, ADFI, and G:F.

On d 14 and 28, one weaned piglet per pen, weighting closest to the average weight for each pen (six piglets per treatment with three female and three male), was chosen for blood sampling after fasting overnight. Blood samples were collected from the anterior vena cava into vacutainer tubes containing EDTAK_2_ (Sanli Medical Technology Development Co. LTD, Liuyang, China) and no anticoagulant to obtain whole blood and serum, respectively. The whole blood was assayed for hematological parameters within 1 hour after sampling. Serum was separated by centrifugation for 10 min at 3,000×g and 4°C, then stored at −20°C until analysis.

After blood sampling, piglets were weighed, then electrically stunned and killed by exsanguination to obtain intestinal and organ tissues. The heart, liver, spleen, lung, and kidney were weighed to calculate the organ index. Organ and intestinal (middle sections of duodenum, jejunum, and ileum) tissue samples were aseptically isolated, flushed with 0.9% salt solution, then fixed in 10% formaldehyde-phosphate buffer, and finally kept at 4°C for microscopic assessment.

### Chemical analysis of feed

Ingredients and diets were analyzed according to AOAC [21] procedures including crude protein, total phosphorus, and calcium. For the analysis of most amino acids, ingredients and diets were hydrolyzed in 6 *N* HCl at 110°C for 24 hours. The sulfur amino acid content was measured after performic acid oxidation (AOAC) [21]. Amino acid analyses were performed using High Performance Liquid Chromatography (Hitachi L-8800 Amino Acid Analyzer, Tokyo, Japan).

### Hematological and serum biochemical parameters analysis

The hematological parameters were determined using a Sysmex Microcell Counter CT-180 (Tokyo, Japan) and included white blood cells, red blood cells, hemoglobin, hematocrit, mean corpuscular volume, mean corpuscular hemoglobin, mean corpuscular hemoglobin concentration, red cell distribution width, platelets, mean platelet volume, platelet distribution width, neutrophils, eosinophils, basophils, lymphocytes, and monocytes.

Serum biochemical parameters in experiment 3 included total protein, albumin, glucose, triglyceride, total cholesterol, creatinine, urea nitrogen, alanine aminotransferase, aspartate aminotransferase, and alkaline phosphatase were measured using commercially-available kits (BioSino Biotechnology and Science Incorporated Beijing, China) using an Automatic Biochemical Analyzer (Hitachi 7160, High Technologies Corporation, Tokyo, Japan). These metabolites were included to cover a wide range of toxicities with possible effects on electrolyte balance, metabolism (carbohydrates, protein, fat, and minerals), and damage to the major organ systems.

### Histopathology analysis

The tissue samples were fixed in 10% buffered formalin for 24 h and then treated following dehydration, clearing and paraffin embedding procedures. Paraffin sections of 5 μm thickness were stained with hematoxylin and eosin. The histopathologic evaluation of organs was accessed according to the pathological scoring standards [22].

### Statistical analysis

In experiment 1, each pen was considered as the experimental unit. Significant differences between data were analyzed using paired *t*-test in SAS 9.2 (SAS, Institute, Cary, NC, USA) statistical software, and differences were considered statistically significant at p<0.05.

The data from experiment 2 and 3 were analyzed as a ran domized complete block design using the general linear model procedure of SAS 9.2 (SAS Institute, USA). Each pen was considered as the experimental unit. Statistical significance was assessed by analysis of variance. Data from experiment 2 and 3 were analyzed by using orthogonal polynomial contrast. Significance was taken at p<0.05 and tendency at 0.05≤p<0.10.

A probability level of p <0.05 was considered statistically significant. In experiment 2, ADG and ADFI during phase I, II and the whole period (0 to 28) was analyzed by a quadratically fitted line model (y = a×x^2^+b×x+c, the maximum quadratic = −b/[2a]), respectively. Data in experiment 2 was subjected to the nonlinear regression procedures of GraphPad Prism (GraphPad Prism 7.0, GraphPad Software, San Diego, CA, USA).

## RESULTS

### Experiment 1

Compared to control group (Table 2), dietary supplementation of 150 mg/kg sucralose was consumed significantly more (p<0.05) than the control diet during d 1, 4, 7, and the entire experimental period (d 1 to 10). Accordingly, the diet preference percentage of sucralose treatment groups was higher (p< 0.05) than control group on day 1, 4, 7, and the entire experimental period (d 1 to 10).

### Experiment 2

**Figure 1:**
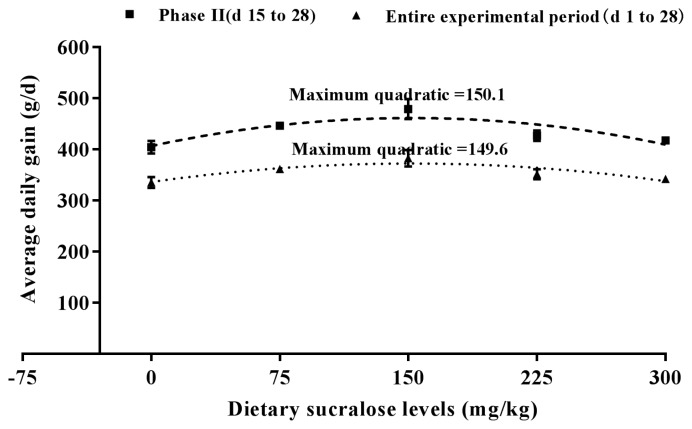
Fitted quadratic plot of average daily gain as a function of dietary sucralose for weaned piglets (experiment 2). The optimal dietary sucralose for phase I was 146.7 mg/kg (y = −0.0008x^2^+0.2347x+265.86, R^2^ = 0.91, p<0.01), and the SE for the estimated parameters of a (−0.0008), b (0.2347), c (265.86) were 0.0005, 0.1545, 9.783, respectively. The optimal dietary sucralose for phase II was 150.1 mg/kg (y = −0.0024x^2^+0.7205x+406.70, R^2^ = 0.74, p<0.01), and the SE for the estimated parameters of a (−0.0024), b (0.7205), c (406.70) were 0.0006, 0.1890, 11.96, respectively. The optimal dietary sucralose for entire experiment period was 149.6 mg/kg (y = −0.0016x^2^+0.4788x+336.21, R^2^ = 0.79, p<0.01), and the SE for the estimated parameters of a (−0.0016), b (0.4788), c (336.21) were 0.0005, 0.1569, 9.936, respectively. SE, standard error.

**Figure 2:**
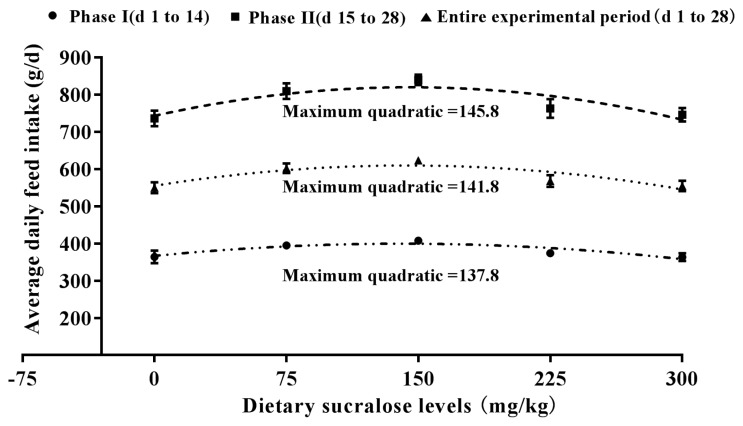
Fitted quadratic plot of average daily feed intake as a function of dietary sucralose for weaned piglets (experiment 2). The optimal dietary sucralose for phase I was 137.8 mg/kg (y = −0.0017x^2^+0.4684x+366.69, R^2^ = 0.81, p<0.01), and the SE for the estimated parameters of a (−0.0017), b (0.4684), c (366.69) were 0.0005, 0.1545, 9.783, respectively. The optimal dietary sucralose for phase II was 145.8 mg/kg (y = −0.0036x^2^+1.0497x+744.09, R^2^ = 0.78, p<0.01), and the SE for the estimated parameters of a (−0.0036), b (1.0497), c (744.69) were 0.0010, 0.3169, 20.06, respectively. The optimal dietary sucralose for entire experiment period was 141.8 mg/kg (y = −0.0027x^2^+0.7655x+555.74, R^2^ = 0.78, p<0.01), and the SE for the estimated parameters of a (−0.0026), b (0.7655), c (555.74) were 0.00068, 0.2114, 13.38, respectively. SE, standard error.

The effect of graded levels of sucralose on the growth performance of weaned piglets is shown in Table 3. The ADG of pigs in 150 mg sucralose treatment group was significantly higher (p<0.05) than the other groups during phase II (d 15 to 28) and the entire experimental period (d 0 to 28). Compared to 0 (control group), 225 and 300 mg dietary sucralose treatment groups, dietary supplementation with 150 mg sucralose significantly increased ADFI during phase I (d 0 to 14), phase II (d 15 to 28) and the entire experimental period (d 0 to 28). Dietary Supplementation with sucralose could improve ADFI. However, when the included level of sucralose was over 150 mg/kg, ADFI was reduced. No significant difference in G:F was found among treatments during any experimental period. According to a fitted quadratic plot model in Figure 1, optimal sucralose inclusion level to maximum ADG was 146.7 mg/kg during phase I, 150.1 mg/kg in phase II, and 149.6 mg/kg during the entire experiment period (d 0 to 28). According to (Figure 2), the quadratic plot model on ADFI indicated that the optimal dosages of sucralose in weaned piglets were 137.8 mg/kg in phase I, 145.8 mg/kg in phase II, and 141.8 mg/kg during the entire experiment period (d 0 to 28), respectively.

### Experiment 3

**Figure 3:**
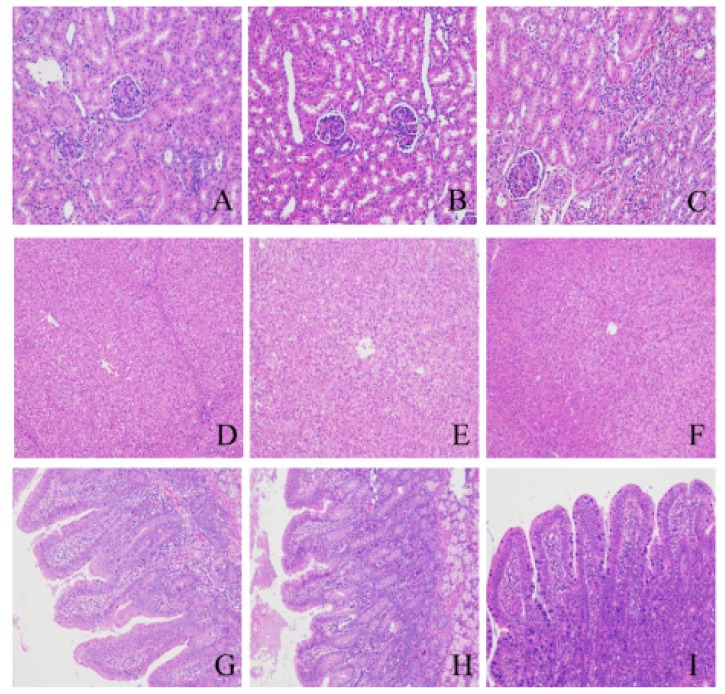
Histopathological examination of the kidney (H&E, 200×), liver and intestinal (H&E, 100×). (A), (B), and (C) were histopathological examinations of the kidney of pigs fed the basal diet and basal diet with 150 or 1,500 mg/kg sucralose, respectively. (D), (E), and (F) were histopathological examinations of the liver of pigs fed the basal diet, and basal diet with 150 or 1,500 mg/kg sucralose, respectively. (G), (H), and (I) were histopathological examinations of the small intestinal (duodenum) of pigs fed the basal diet, and basal diet with 150 or 1,500 mg/kg sucralose, respectively. H&E, haematoxylin and eosin.

In experiment 3, piglets fed diet supplemented with 150 mg/kg sucralose had higher ADG and ADFI than that of pigs in the control group and 1,500 mg/kg treatment groups during the experiment period (Table 4). Hematological parameters (Table 5), serum biochemical parameters (Table 6), and organ index (Table 7) were not significantly different among all treatment groups. Figure 3 shows the histological structures of liver, kidney and small intestines observed in all pigs fed the different diets for 28 days. No significant changes were observed in histopathological analysis of tissues compared to control group.

## DISCUSSION

Most mammals have a preference for sweet feed [23]. Among those with a sweet preference, pigs prefer sweet substances because they have large number of taste buds in the tongue [24]. In the present study, preference tests showed that weaned piglets prefer the diet supplemented with sucralose, and dietary supplementation with 150 mg/kg sucralose could significantly increase feed intake.

Weaned piglets have a preference for a sweet taste, espe cially sucrose [12]. Sucrose is widely used as sweetener in feed. However, Guzmán-Pino et al [25] reported that excessive calories ingested from a sucrose solution may result in decreased feed intake, and further reducing weight gain of weaned piglets. Sucralose is a non-caloric sweetener that is synthesized by selective chlorination of sucrose at three of the primary hydroxyl groups, involving inversion of configuration at carbon-4, from the gluco-to the galactoanalogue [26]. Meanwhile, sucralose has a sweetness potency of about 600 times that of sucrose [15], thus adding a small amount of sucralose into the feed can achieve a similar effect to the sweetness of sucrose.

There have been many reports of sweeteners fed to pigs, but growth performance results are inconsistent. Sweeteners like neohesperidin dihydrochalcone (trade name Sucram) [4] and neotame have been reported to improve the performance of pigs. Adding the artificial sweetener, neotame, in the feed can improve palatability and feed intake for piglets over 35 d [7]. The supplement of sucralose can improve ADG and ADFI of weaned piglets during phase I (d 0 to 14) and phase II (d 15 to 28) in the present study. The present study showed that over 28 d optimum growth rate and feed intake were supported on diets containing 149.6 and 141.8 mg/kg sucralose, respectively. Experiment 2 mainly focused on the efficacy evaluation and to determine the suitable inclusion level in diets. The result found a clear reduction of ADFI when sucralose was dosed over 150 mg/kg. The growth performance (ADG and ADFI) started to decrease when 225 and 300 mg/kg sucralose was included in diets. It was the decreasing the palatability of the diet that further reduced ADFI and ADG, not that sucralose had adverse effects on health of weaned piglets. However, several researches on other sweeteners such as sorbitol [27] and stevia [28] suggested that no beneficial effects on pigs were observed. These responses are likely to be affected by the age and weight of the pig at weaning and by the composition of the basal diet. The stevia-containing diets did not increase feed intake of weaned piglets [28]. Similarly, there were no effects of the dietary sorbitol levels were observed on growth performance [27]. The positive effects of sucralose were observed both during phase I (d 0 to 14) and phase II (d 15 to 28) in the present study. However, some studies [4,29] found that pigs required a certain period of time before positive effects of sweeteners on their performance could be observed.

The safety of sweeteners has been controversial [30]. Thus, the safety assessment of sucralose is an important aspect before its application as a feed additive. Experiment 3 mainly focused on tolerance evaluation and ensuring whether the suitable inclusion level of 150 mg/kg sucralose is safe for weaned piglets. If ten-fold suitable level of sucralose showed no indication of toxicity in weaned pigs, then it means that weaned pigs could tolerate ten-fold suitable level of sucralose. In other words, the recommended suitable level of 150 mg/kg is safe for weaned piglets [31]. The safety of sucralose has been the subject of rigorous and extensive investigation. The maximum tolerable doses were due to poor palatability of high doses of sucralose, not toxicity [14]. This study demonstrated that sucralose has negative effect on ADFI of weaned piglets when including level is over 150 mg/kg in diet. Grice and Goldsmith [14] also reported that 30 g/kg/d sucralose could reduce feed intake in rats. This may be that a high level of sucralose decreases palatability of diet. In the present study, dietary supplementation of sucralose up to 1,500 mg/kg had no detrimental effects on hematological parameters, biochemical parameters and organ index, which are in accordance with studies in the rat and dogs. Goldsmith et al [32] reported that dietary sucralose levels of up to 25 g/kg/d had no adverse effects on biochemical parameters, organ index and hematological parameters in rats. They also found that administration of 1,500 or 3,000 mg/kg/d sucralose had no sucralose-related toxicity effects in rats. Additionally, a 12-month study in dogs also revealed that dietary supplementation with sucralose up to 30 g/kg/d had no adverse effects on the hematological and clinical chemistry assessed [32]. Otherwise, the studies for other sweeteners such as neotame in weaned piglets obtained similar results, no significant differences on hematological parameters and organ index were observed with dietary supplemented levels up to 500 mg/kg [7]. Hematological parameters play an important role in evaluating the physiological state and injuries caused by certain substances [6]. Serum biochemical parameters reflect the health status of the kidney and liver functions and lipid metabolism [33]. Serum alanine aminotransferase and aspartate aminotransferase are commonly measured as clinical biomarkers for liver health [33]. In the present study, sucralose was found to be safe when fed to weaned piglets at a dose of 1,500 mg/kg, which is an estimated 10-fold higher dose than the commended optimal dose for weaned piglet. Therefore, the highest dose did not induce noticeable signs of toxicity.

## CONCLUSION

This study indicated that dietary supplemental of sucralose may increase growth performance of weaned piglets. The concentrations of sucralose over 150 mg/kg may decrease palatability of feed and further reduce feed intake. Our results also showed that pigs could tolerate up to 1,500 mg/kg sucralose without adverse effect on their health. Therefore, supplementing 150 mg/kg sucralose for weaned piglets is recommended in this study.

## Figures and Tables

**Table 1:** Ingredient and analyzed composition of basal diets (as-fed basis, g/kg)1)

Items	Basal diet	

	Phase I	Phase II
Ingredient		
Ground corn	533.8	581.6
Soybean meal (46% crude protein)	177.5	159.0
Expanded soybean	163.5	130.0
Dried whey (12% crude protein)	46.4	62.1
Fish meal	27.4	26.5
Soybean oil	16.9	10.2
Dicalcium phosphate	11.2	10.0
Ground limestone	7.4	5.0
Salt	2.3	3.0
L-Lysine HCl	3.5	3.8
L-Threonine	1.3	1.2
DL-Methionine	1.8	1.6
Choline chloride	2.0	1.0
Vitamin-mineral premix2)	5.0	5.0
Chemical analysis		
Digestible energy3) (MJ/kg)	14.9	14.7
Crude protein	209.9	193.3
Lysine	14.6	13.8
Methionine	4.4	3.9
Methionine+cysteine	7.4	6.8
Threonine	7.9	7.7
Calcium	8.5	8.2
Total phosphorus	6.6	6.0

1) In experiment 1, the basal diet was supplemented with 150 mg/kg sucralose. In experiment 2, the basal diets were supplemented with 75, 150, 225, or 300 mg/kg sucralose (Phase I and II). In experiment 3, the basal diets were supplemented with 150, or 1,500 mg/kg sucralose (Phase I and II).

2) Provided per kg of diet: vitamin A, 12,000 IU; vitamin D_3_, 2,000 IU; vitamin E, 30 IU; vitamin K_3_, 2.5 mg; thiamin, 2.5 mg; riboflavin, 4 mg; pyridoxine, 3 mg; vitamin B_12_, 20 μg; niacin, 40 mg; pantothenic acid, 12.5 mg; folic acid, 0.7 mg; biotin, 0.07 mg; Fe as iron sulfate, 100 mg; Cu as copper sulfate, 90 mg; Zn as zinc sulfate, 80 mg; Mn as manganese sulfate, 30 mg; I as potassium iodide, 0.25 mg; Se as sodium selenite, 0.15 mg.

3) Calculated value according to DE value of each ingredient provided by NRC [19].

**Table 2:** The effects of dietary sucralose on diet preference of weaned piglets (experiment 1)

Items	Dietary sucralose level (mg/kg)		SEM	p-value

	0	150		
Diet preference percentage (%)				
d 1	44.9	55.1	2.64	0.007
d 2	50.4	49.6	4.19	0.848
d 3	48.7	51.3	2.96	0.415
d 4	45.3	54.7	3.65	0.365
d 5	48.4	51.6	2.05	0.165
d 6	48.2	51.8	1.55	0.054
d 7	45.2	54.8	3.61	0.034
d 8	47.9	52.1	4.10	0.351
d 9	45.7	54.2	5.91	0.197
d 10	48.2	51.8	4.46	0.435
Average diet preference percentage (d 1 to 10)	47.3	52.6	3.73	0.005

Data are the means of 8 replications.

SEM, pooled standard error of the mean.

**Table 3:** The effects of graded levels of sucralose on the performance of weaned piglets (experiment 2)

Items	Dietary sucralose level (mg/kg)					SEM	p-value		
	
	0	75	150	225	300		ANOVA	Linear	Quadratic
0 d (kg)	7.92	7.92	7.94	7.98	7.99	0.17	0.998	0.734	0.933
14 d (kg)	11.74	11.86	11.96	11.80	11.73	0.22	0.942	0.897	0.434
28 d (kg)	17.41	18.11	18.74	17.71	17.58	0.27	0.005	0.989	0.001
Phase I (d 0 to 14)									
Average daily gain (g/d)	268	278	287	276	267	5.33	0.049	0.816	0.004
Average daily feed intake (g/d)	364	395	408	374	363	10.21	0.016	0.500	0.002
Feed efficiency (gain to feed ratio)	0.74	0.70	0.70	0.74	0.74	0.02	0.160	0.510	0.058
Phase II (d 15 to 28)									
Average daily gain (g/d)	406	447	480	426	418	5.93	<0.001	0.866	<0.001
Average daily feed intake (g/d)	737	810	840	763	747	20.43	0.006	0.689	0.001
Feed efficiency (gain to feed ratio)	0.55	0.55	0.57	0.56	0.56	0.01	0.584	0.322	0.619
Entire experiment (d 0 to 28)									
Average daily gain (g/d)	336	362	384	351	342	4.75	<0.001	0.962	<0.001
Average daily feed intake (g/d)	551	603	624	569	555	13.47	0.002	0.577	0.001
Feed efficiency (gain to feed ratio)	0.61	0.60	0.61	0.62	0.62	0.01	0.517	0.223	0.542

Data are the means of 6 replications.

SEM, pooled standard error of the mean; ANOVA, analysis of variance.

**Table 4:** The effects of graded levels of sucralose on the performance of weaned piglets (experiment 3)

Items	Dietary sucralose level (mg/kg)			SEM	p-value		
	
	0	150	1,500		ANOVA	Linear	Quadratic
0 d (kg)	7.93	7.96	8.03	0.18	0.918	0.683	0.947
14 d (kg)	12.10	12.67	12.25	0.20	0.137	0.729	0.051
28 d (kg)	17.05	18.61	17.42	0.29	0.001	0.418	<0.001
Phase I (d 0 to 14)							
Average daily gain (g/d)	292	336	301	4.48	<0.001	0.092	<0.001
Average daily feed intake (g/d)	385	450	395	16.32	0.043	0.951	0.014
Feed efficiency (gain to feed ratio)	0.76	0.75	0.74	0.02	0.790	0.577	0.684
Phase II (d 15 to 28)							
Average daily gain (g/d)	355	423	369	9.00	<0.001	0.194	<0.001
Average daily feed intake (g/d)	730	852	747	26.76	0.012	0.308	0.005
Feed efficiency (gain to feed ratio)	0.49	0.50	0.49	0.01	0.941	0.739	0.912
Entire experiment (d 0 to 28)							
Average daily gain (g/d)	323	379	335	4.78	<0.001	0.052	<0.001
Average daily feed intake (g/d)	558	651	579	13.26	<0.001	0.287	<0.001
Feed efficiency (gain to feed ratio)	0.58	0.58	0.58	0.01	0.943	0.735	0.968

Data are the means of 6 replications.

SEM, pooled standard error of the mean; ANOVA, analysis of variance.

**Table 5:** The effects of graded levels of sucralose on hematological parameters of weaned piglets (experiment 3)

Items	Dietary sucralose level (mg/kg)			SEM	p-value		
	
	0	150	1,500		ANOVA	Linear	Quadratic
White blood cells (10^9^/L)	18.38	22.65	15.85	2.43	0.169	0.168	0.188
Red blood cells (10^12^/L)	6.19	5.97	6.32	0.21	0.524	0.417	0.432
Hemoglobin (g/L)	100.17	100.50	104.67	3.69	0.639	0.352	0.982
Hematocrit (%)	0.38	0.38	0.40	0.01	0.587	0.303	0.847
Mean corpuscular volume (%)	62.05	64.17	63.22	1.46	0.601	0.880	0.326
Mean corpuscular hemoglobin (%)	16.18	16.83	16.63	0.42	0.541	0.735	0.298
Mean corpuscular hemoglobin concentration (%)	261.00	262.17	262.67	2.96	0.920	0.751	0.805
Red cell distribution width (%)	21.73	21.17	20.25	0.75	0.389	0.196	0.684
Platelet (10^9^/L)	495.67	479.17	453.67	47.07	0.819	0.554	0.849
Mean platelet volume (fL)	12.00	11.60	12.15	0.47	0.703	0.593	0.526
Platelet distribution width (%)	15.63	15.00	12.77	1.12	0.196	0.078	0.821
Neutrophils (10^9^/L)	2.26	3.20	1.00	1.32	0.513	0.325	0.558
Eosinophils (10^9^/L)	0.20	0.20	0.26	0.55	0.565	0.294	0.943
Basophils (10^9^/L)	1.17	2.73	1.40	0.75	0.310	0.652	0.149
Lymphocyte (%)	74.97	69.28	78.40	6.21	0.589	0.458	0.483
Monocyte (%)	5.02	5.37	5.15	0.44	0.583	0.979	0.579

Data are the means of 6 replications.

SEM, pooled standard error of the mean; ANOVA, analysis of variance.

**Table 6:** The effects of graded levels of sucralose on serum biochemical parameters of weaned piglets (experiment 3)

Items	Dietary sucralose level (mg/kg)			SEM	p-value		
	
	0	150	1,500		ANOVA	Linear	Quadratic
Total protein (g/L)	42.47	40.50	44.53	3.17	0.675	0.469	0.619
Albumin (g/L)	25.70	24.15	25.72	2.05	0.826	0.794	0.582
Glucose (mmol/L)	5.59	5.30	6.30	0.52	0.404	0.217	0.613
Triglyceride (mmol/L)	0.47	0.45	0.43	0.06	0.908	0.732	0.793
Total cholesterol (mmol/L)	1.78	1.59	1.93	0.17	0.407	0.303	0.394
Creatinine (μmol/L)	66.15	58.53	72.50	7.28	0.419	0.303	0.414
Urea nitrogen (mmol/L)	2.21	1.91	2.98	0.37	0.144	0.069	0.462
Alanine aminotransferase (U/L)	30.97	27.93	29.77	3.22	0.801	0.985	0.513
Aspartate transaminase (U/L)	31.77	25.32	40.67	7.27	0.350	0.213	0.466
Alkaline phosphatase (U/L)	269.57	240.58	180.02	26.20	0.078	0.029	0.579

Data are the means of 6 replications.

SEM, pooled standard error of the mean; ANOVA, analysis of variance.

**Table 7:** The effects of graded levels of sucralose on organ index of weaned piglets (g/kg body weight, experiment 3)

Items	Dietary sucralose level (mg/kg)			SEM	p-value		
	
	0	150	1,500		ANOVA	Linear	Quadratic
Heart	5.43	5.16	5.23	0.22	0.678	0.742	0.421
Liver	31.95	31.27	31.12	1.59	0.926	0.786	0.782
Spleen	2.44	2.29	2.57	1.59	0.619	0.437	0.562
Lung	11.53	10.99	10.82	0.54	0.639	0.483	0.534
Kidney	2.58	2.50	2.62	0.10	0.701	0.563	0.548

Data are the means of 6 replicates.

SEM, pooled standard error of the mean; ANOVA, analysis of variance.
